# The *FTO* genetic variants are associated with dietary intake and body mass index amongst Emirati population

**DOI:** 10.1371/journal.pone.0223808

**Published:** 2019-10-17

**Authors:** Maha Saber-Ayad, Shaista Manzoor, Hadia Radwan, Sarah Hammoudeh, Rahaf Wardeh, Ahmed Ashraf, Hussein Jabbar, Rifat Hamoudi

**Affiliations:** 1 College of Medicine, University of Sharjah, Sharjah, UAE; 2 Research Institute for Medical and Health Sciences, University of Sharjah, Sharjah, UAE; 3 College of Medicine, Cairo University, Cairo, Egypt; 4 Clinical Nutrition and Dietetics Department, College of Health Sciences, University of Sharjah, Sharjah, UAE; Texas A&M University College Station, UNITED STATES

## Abstract

**Background:**

The risk of obesity is determined by complex interactions between genetic and environmental factors. Little research to date has investigated the interaction between gene and food intake. The aim of the current study is to explore the potential effect of fat mass and obesity-associated protein gene *(FTO) rs9939609* and *rs9930506* single nucleotide polymorphism (SNP) on the pattern of food intake in the Emirati population.

**Methods:**

Adult healthy Emirati subjects with Body mass index (BMI) of 16–40 kg/m^2^ were included in the study. Genotyping for *FTO rs9939609*(A>T) and *rs9930506*(A>G) was performed using DNA from saliva samples. Subjects were categorized according to the WHO classification by calculating the BMI to compare different classes. Dietary intake was assessed by a sixty-one-item FFQ that estimated food and beverage intakes over the past year. The daily energy, macronutrient, and micronutrient consumption were computed.

**Results:**

We included 169 subjects in the final analysis (mean age 30.49± 9.1years, 57.4% females). The mean BMI of the study population was 26.19 kg/m^2^. Both SNPs were in Hardy Weinberg Equilibrium. The *rs9939609* AA genotype was significantly associated with higher BMI (p = 0.004); the effect was significant in females (p = 0.028), but not in males (p = 0.184). Carbohydrate intake was significantly higher in AA subjects with a trend of lower fat intake compared to other genotypes. The odds ratio for the AA was 3.78 in the fourth quartile and 2.67 for the A/T in the second quartile of total carbohydrate intake, considering the first quartile as a reference (95% CI = 1.017–14.1 and 1.03–6.88, respectively). Fat intake was significantly lower in the *FTO rs9930506* GG subjects. The presence of *FTO rs9930506* GG genotype decreased the fat intake in subjects with *FTO rs9939609* AA (p = 0.037).

**Conclusions:**

The results of this study highlight the interaction of the FTO risk alleles on the food intake in Emirati subjects. The *FTO rs9939609* AA subjects had higher carbohydrate and lower fat intake. The latter was accentuated in presence of *rs9930506* GG genotype.

## Introduction

The consequences of the obesity epidemic have been a great burden on the health systems worldwide; including an increased risk of serious chronic conditions; such as heart diseases, cancer, and diabetes [1]. The interplay between the environmental changes and the genetic factors has led to a significant increase in obesity prevalence worldwide [2]. Gene-environment interaction is defined as a response or adaptation to an environmental agent, a behavior, or a change in behavior, conditional to the genotype of the individual [3]. Such interaction can give new insight into the variation of body mass index (BMI) and obesity susceptibility among individuals [4]. Mathematical models have predicted that even a small energy excess or deficit (around 1%) may result over time in weight gain or loss [5]. Obviously, environmental differences may mask the genetic effect on BMI [6].

The fat mass and obesity-associated *(FTO)* gene [chromosome 16 (16q12.2a)] has shown the largest effect on BMI, although the increase is modest [7]. The link of *FTO rs9939609* A allele to high BMI was described in many previous studies all over the world [8,9] and in the Middle East; including Saudi Arabians [10], Kuwaitis [11], Emiratis [12] and diabetic Palestinians [13]. It has been also identified as a genetic risk of metabolic syndrome in Egyptians [14]. The *FTO rs9930506* (G>A) is the most strongly linked neighboring SNP to *rs9939609* and was reported to be highly associated with a high BMI, especially in European Americans and Hispanic Americans who showed strong links[9]. Homozygotes of the “G” allele of this SNP experienced an additional 1.3 BMI units compared to homozygotes of the common “A” allele [15].

The UAE is at the top of the list of countries with high obesity prevalence [16]. The prevalence has dramatically increased in the last few decades due to the changing lifestyle and eating habits[17]. In the current study, the aim is to explore the potential effect of two *FTO* SNPs *rs9939609* and *rs9930506*, strongly linked to obesity, on the pattern of food intake in the Emirati population. We hypothesize that those SNP’s are affecting predilection to certain types of food, that leads to more significant weight gain.

## Subjects and methods

### Subjects

This study is a cross-sectional study of two *FTO* SNPs. The sample size was calculated according to the following formula: S = [(1.96)^2^ p (1-p)] / d^2^, where p = expected prevalence of *FTO* SNPs in the population based on previous studies, and d = absolute error or precision (i.e. the difference between the calculated prevalence and the true prevalence). This formula applies for a type I error of 5% (p<0.05 is considered statistically significant).

We recruited healthy adult Emirati subjects from the University of Sharjah and primary health care centers.

Our inclusion criteria are adult healthy Emirati subjects, competence to give an informed consent and to complete the questionnaire. Exclusion criteria are body mass index (BMI) below 16 or above 40 kg/m^2^, inability to give a consent or to complete the questionnaire.

Ethical approval was obtained before the study started. All participants gave informed consent according to the study protocol approved by the Research and Ethics Committee, University of Sharjah. We excluded subjects with hypertension, diabetes mellitus, and other chronic diseases. All were non-smokers and do not drink alcohol. Subjects who followed strict dietary changes in the past 2 years were also excluded. We made sure that the participants did not eat before 30 minutes of collecting 2 ml of saliva samples. They were asked to give saliva without phlegm. The samples were preserved at -20 C^0^ and DNA extraction using the QIAamp extraction kit (cat# 51306) was performed within 7 days.

### Anthropometry

Anthropometric measurements were taken using standardized techniques and calibrated equipment. Participants were weighed to the nearest 0.1 kg wearing light clothing. Using a stadiometer, height was measured without shoes and recorded to the nearest 0.5 cm. BMI was calculated as weight in kilograms divided by the square of height in meters (kg/m^2^). BMI was categorized according to the WHO classification: BMI less than 18.5 kg/m^2^ as underweight, BMI 18.5 to 24.9 kg/m^2^ as normal weight, BMI 25.0 to 29.9 kg/m^2^ as overweight, and BMI 30.0 kg/m^2^ or greater as obese [18]. BMI was also expressed in quartiles for further analysis.

### Dietary survey

Dietary intake was assessed by a sixty-one-item FFQ that estimated food and beverage intakes over the past year [19]. It included information on consumption of commonly consumed food items and beverages in the UAE. The subjects were asked to record the frequency of consumption either per day, per week, per month, per year or never. Each listed food item had a standard portion, expressed in household measures. A reference portion, representing one standard serving expressed in household measures, was defined for each food item. Participants were assisted with the reference portions of the two-dimensional food portion visual (Millen and Morgan, Nutrition Consulting Enterprises, Framingham, Massachusetts, United States), as well as supplementary visual aids about portion sizes of common items in the traditional Gulf and Middle Eastern cuisine meals [*Abu Dhabi Food Control Authority*. *A Photographic Atlas of Food Portions for the Emirate of Abu Dhabi*. *User's Guide*. *Abu Dhabi*: *2014*. *Abu Dhabi Food Control Authority*] to help to estimate ingested quantities. The reported frequency of each food item and beverage was then converted to a daily portion intake. The daily energy, macronutrient, and micronutrient consumption by participants were computed using the food composition tables provided by the NUTRITIONIST PRO^TM^ diet analysis software (Axxya Systems LLC., USA, version 5.1.0, 2014, First Fata Bank, Nutritionist Pro, San Bruno, CA).

### Genotyping

Genotyping for *FTO rs9939609* (A>T) and *rs9930506 (*A>G) was performed as described in our previous study [20]; using StepOne Real-Time PCR Systems (Thermo Fischer Scientific, USA) using TaqMan® Drug Metabolism Genotyping Assay (Applied Biosystems, USA). Context sequence is shown in Box 1. Allele-1 (wild) is bound to VIC, allele-2 is bound to FAM. We used the Chi-square test through the online tool http://www.oege.org/software/hwe-mr-calc.shtml; to estimate Hardy–Weinberg equilibrium and the allele frequency [21].

**Box 1. Context Sequence of *FTO rs9939609* (A>T) and *rs9930506 (*A>G)**

**NCBI reference | Context sequence**

***rs9939609 |*** GGTTCCTTGCGACTGCTGTGAATTT[A/T]GTGATGCACTTGGATAGTCTCTGTT

***rs9930506 |*** AGGGACACAAAAAGGGACATACTAC[A/G]TGAATTACTAATATCTAAGAAAATA

### Statistical analysis

We described data in terms of mean±standard deviation (SD), frequencies (number of cases) and percentages when appropriate. Categorical data were compared using Chi-square (X^2^). Independent-samples t-test was used to compare the homozygous risk genotype group to other genotypes for each SNP. The odds ratio was used to describe the effect size when there is a significant difference. Correlation between various continuous variables and when significant, multiple regression was used. p-value≤0.05 was considered statistically significant. All statistical calculations were done using computer program SPSS (Statistical Package for the Social Science; SPSS Inc., Chicago, IL, USA) version 23 for Microsoft Windows.

## Results

In the current study, we initially recruited 215 healthy adult Emiratis. We excluded 10 subjects with a BMI above 40 kg/m^2^ and 9 subjects below 16 kg/m^2^; 27 subjects were further excluded due to extreme values provided for any single food item. The data of only 169 subjects were considered for further analysis. The normality of data was checked by QQ-plot. **Table 1** shows the baseline characteristics of the study group. Mean age of the study population was 30.49± 9.1 years, range 18–54 years, 57.4% females. The mean BMI of the population was 26.19 kg/m^2^, which indicates overweight. Males had higher mean BMI as compared to females (25.65 and 26.90 kg/m^2^, respectively).

**Table 1:** Participant characteristics.

		Mean ± SD (range)	
Age (years)		30.49 ± 9.19 (18–54)	
Gender			
	Male (n,%) Female (n,%)	97 (57.4%) 72 (42.6%)	
Total carbohydrate intake (g/d)		395.46 ± 142.73 (113.49–811.29)	
Total protein intake (g/d)		150.60 ±58.12 (37.12–346.59)	
Total fat intake (g/d)		126.45 ±56.52 (30.48–296.10)	
BMI (Kg/m^2^)		26.19 ± 4.63 (17.58–37.11)	
BMI (n,%)			
	BMI<24.9	69 (40.8%)	
	BMI = 25–29.9	60 (35.5%)	
	BMI = 30 or more	40 (23.7%)	
*FTO rs9939609* (n,%)			
	A/A	27 (16.0%)	
	A/T	76 (45.0%)	
	T/T	66 (39.1%)	
*FTO rs9930506* (n,%)	*		
	G/G	35 (20.7%)	
	A/G	74 (43.8%)	
	A/A	59 (34.9%)	

*168 subjects were genotyped for *FTO rs9930506*.

Both SNPs were in Hardy-Weinberg equilibrium (using Chi square test, the p-value = 0.52 for *rs9939609* and 0.19 for *rs9930506*). Minor allele frequency was 0.38 for *rs9939609* and 0.43 for *rs9930506*. The frequencies of BMI quartiles and different genotypes in male and female participants are presented in Table 2. BMI significantly correlated with age (Pearson correlation = 0.308, p = 0.0001). With every year increase in age, there is 0.156 kg/m^2^ increase in BMI.

**Table 2:** Carbohydrate, protein and fat intake according to *FTO rs9939609* and *rs9930506*.

Food category (mean in g/day±SD)	*FTO rs9939609*		df*	p	*FTO rs9930506*		df*	p
	A/A (n = 27)	Others (n = 142)			G/G (n = 35)	Others (n = 134)		
Carbohydrates	447.57±163.03	385.55± 136.95	33.334	0.038*	436.32±156.29	384.78 ±137.60	48.653	0.057
Protein	161.30 ±66.10	148.56±56.50	33.606	0.298	162.11±68.87	147.56 ±54.86	45.88	0.189
Fat	109.64±54.08	129.65±56.58	37.648	0.092	107.69±50.69	131.35±57.10	58.619928	0.027*

*denotes p-value <0.05

### Association of SNPs to BMI

*The FTO rs9939609* AA genotype, detected in 15.9% of the study population, was significantly associated with high BMI (>25kg/m^2^), (Pearson’s Chi-square p = 0.004, Effect size: Phi = Cramer’s V = 0.257). Females had a significantly higher BMI according to *FTO rs9939609* genotype (p = 0.028), but not males (p = 0.184). This was not observed when comparing *FTO rs9930506 GG* (detected in 20.7%) with others (p = 0.215).

Multinomial logistic regression showed a significant decrease in weight in T/T genotype of *rs993609* with a 0.95 kg/m^2^ decrease in BMI (p = 0.02) in subjects between 25–29.9 kg/m^2^. This effect was not detected in subjects with a BMI of 30 or more.

### Association of SNPs to macronutrient intake

**Fig 1:**
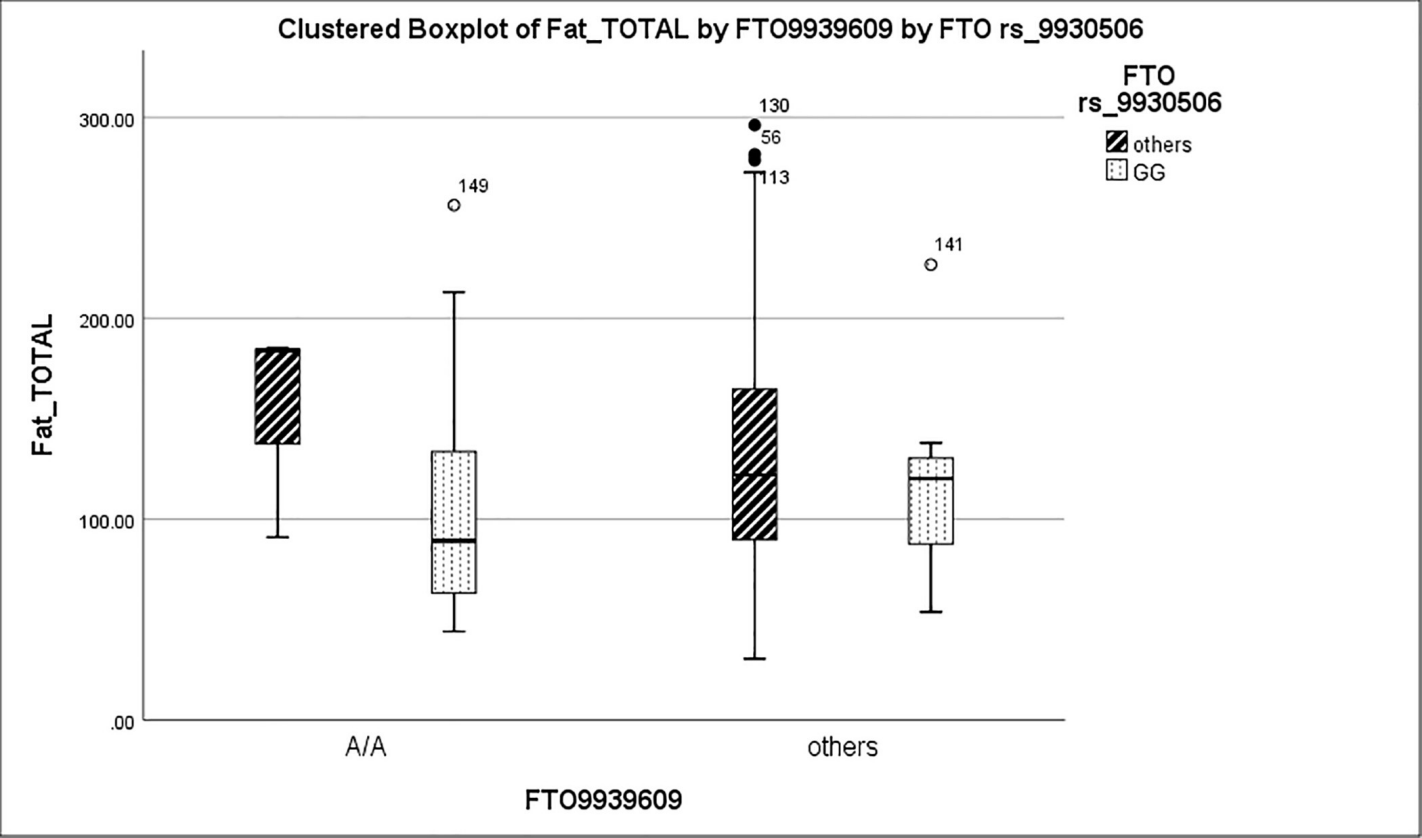
Interaction of *FTO rs9939609* and *rs9930506* on fat intake. In the group of subjects carrying *FTO rs9939609* A/A risk allele (n = 27), we explored the additive effect of *rs 9939506* risk allele G, using independent samples t-test to compare the two subgroups. If homozygous for both risk alleles, the subject intake of fat is significantly lower. The presence of *FTO rs9930506* GG genotype significantly decreased fat intake in subjects with *FTO rs9939609* AA (n = 3 and 24 respectively, p = 0.037, using Mann-Wintney non-parametric test). This was not noticed in other *FTO rs9939609*, (p-value = 0.32).

Carbohydrate intake was significantly higher in the *FTO rs9939609* AA subjects. They also had a trend of higher protein and lower fat intake compared to other genotypes. Fat intake was significantly lower in the *FTO rs9930506* GG subjects and they had a trend of higher carbohydrate and higher protein intake, **Table 2.** We explored the additive effect of *rs9939506* risk allele G. Fat intake was significantly lower in the *FTO rs9930506* GG *rs9939609* AA subjects (subjects homozygous for both risk alleles, n = 3), **Fig 1.**

Quartiles of total carbohydrate intake were compared, setting the first quartile as a reference. The odds ratio for the AA genotype was 3.78 in the fourth quartile and 2.67 for the A/T in the second quartile of total carbohydrate intake, **Table 3.**

**Table 3:** Effect of *FTO rs 9939609* A allele (3 genotypes) on carbohydrate intake.

Tertile of total carbohydrate intake	N	OR	95% CI	P
First*	44	-	-	-
Second	43	2.67 [A/T]	1.03–6.88	0.042*
Third	42	1.51	0.62–3.7	0.36
Fourth	40	3.78 [A/A]	1.017–14.1	0.047*

*First quartile is the reference, OR = Odds ratio, CI = Confidence interval.

We investigated which carbohydrate-rich food items correlated significantly to total carbohydrate intake, in the *FTO rs9939609* AA group compared to other genotypes. White bread, rice, and rice-based products were highly correlated with carbohydrate intake in the AA group, whereas intakes of high carbohydrate with higher fat food items including pies, fried potato, chips were significantly correlated with other genotypes of this SNP.

There was no significant difference between food intake of different macronutrients and BMI.

### Association of SNPs to micronutrient intake

*FTO rs9939609* AA was associated with a significantly higher intake of Vitamin D, B1, B2, B6, and selenium, (p<0.05). Subjects in various BMI quartiles did not differ significantly regarding the intake of vitamins and trace elements. However, there was a significant but weak correlation between BMI and intake of B3 (Pearson = 0.165*, p = 0.032), Calcium (Pearson = 0.180*, p = 0.018), Magnesium (Pearson = 0.193*, p = 0.012) and potassium (Pearson = 0.180*, p = 0.019).

## Discussion

The current study was conducted on the Emirati population to explore the effect of *FTO* variants on food predilection. It provides a distinct effect of the *FTO* risk alleles in Emiratis’ food intake in comparison to that of other ethnicities. Variants of both *rs9939609* and *rs9930506* showed highly significant association with high BMI in the database of The Genetic Investigation of ANthropometric Traits (GIANT), [22]. In our study, the homozygous risk genotype of *rs9939609* and *rs9930506* genotypes, were detected in 16% and 20.7% of the study population. This is close to our previous study that showed a prevalence of 20.5% and 21.9% of those genotypes, respectively in the Emirati population [20].

The wide variation of the *FTO rs9939609* prevalence was observed among several populations, for instance, the minor allele frequency (MAF) was 26.6% in Pakistanis [23], and 42.3% in Russians [24]. In regard to the *FTO rs9939506* prevalence, the MAF was documented as 45% among Europeans [22], compared to 20% in the Chinese population [25].

The *FTO rs9939609* AA genotype was significantly associated with high BMI, in line with other studies [8,9]. Females showed a significant difference in BMI according to genotype, in line with the study of Khan et al., 2018 [12]. In our previous study on a cohort from the National Diabetes Project, we could not detect an association with BMI in the Emirati subjects with the A allele. This may be due to the lower percentage of female subjects in the previous study. Such gender difference was previously described in Swedish and Chinese children and adolescents with obesity [26,27]. However, this was not found in non-Hispanic whites and African Americans [28].

Our study showed that carbohydrate intake was significantly higher in the *FTO rs9939609* AA subjects and they had a trend of higher protein and lower fat intake compared to other genotypes. In their study on gene-environment interactions, Young et al. showed that the diet score with high protein, food weight, and saturated fat showed a strong positive association with BMI. They found that the effect of *FTO* on BMI is enhanced in individuals with a higher diet score [29].

Previous studies showed that obesity susceptibility genes may interact with saturated fatty acids, but not mono- or poly-unsaturated fatty acids, to promote weight gain[30]. As a consequence, high-fat diets, with an enhanced palatability and high energy content, may have a primary role for the obesity epidemic. Moreover, increased intake of refined carbohydrates, and sugar-sweetened beverages, over the past few decades led to an increased prevalence of obesity [31]. In contrast to previous studies, our results showed that the AA allele is associated with higher carbohydrate and lower fat intake [32,33]. It should be noticed that the previous studies were performed on Caucasians. Age may play a role in food preference. The weather difference may explain the predilection to a high-fat diet in Caucasians carrying the risk allele. The results in children may be more robust, as the social desirability and underreporting is probably less than that in adult [34]. However, it is generally difficult to accurately estimate energy intake and expenditure in children [35]. It should be noticed that the environmental changes over time may modify the effect of *FTO* genotype on BMI by modifying the penetrance of genetic risk factors, leading to diverse phenotypes [36]. Such environmental changes may also include micro-nutrients; e.g. Vitamin D was shown to significantly modify the *FTO* effects on weight gain, with a more prominent effect of the genotype among children with insufficient vitamin D levels [37].

Noteworthy, there was a significant correlation between high carbohydrate intake and high-fat items in the A/T and the T/T genotype of *rs9939609* compared to the AA genotype, although the latter was significantly correlated with high carbohydrate lower fat foods. If combined with *rs9939506* G/G genotype of the *rs9939506*, there is significantly less fat intake in the AA genotype group. Such SNP interaction is first to be reported in the current study.

Dietary intake and total energy consumption are one of the major environmental players in obesity. The *FTO* A allele was proved to raise the risk of increasing food intake through impairing central processing of satiety [38], as the *FTO* gene is highly expressed in the hypothalamus [39]. Many studies showed that dietary intake plays a significant role in the development of obesity [40]. The relationship between specific dietary nutrient intake and gene variations on obesity was recently investigated. The high-energy intake has been associated with high consumption of protein, carbohydrate, fat and added sugars [41]. On the other hand, diets high in micronutrients such as vegetables, fruits, and whole grains were inversely related to the prevalence of obesity [42]. There is increasing evidence for the importance of micronutrients in genome stability and health. Even small damages caused by micronutrient deficiencies in the genome can produce serious consequences [30].

The *FTO* is a 505 amino acid protein with Alpha-ketoglutarate-dependent dioxygenase. It repairs alkylated DNA and RNA by oxidative demethylation. In higher eukaryotes, it specifically demethylates N(6)-methyladenosine (m^6^A) RNA, the most prevalent internal modification of messenger RNA (mRNA), [43]. The *FTO* transcripts containing the A (risk) allele of *rs9939609* were more abundant than those with T allele in blood and fibroblasts [44]. Interestingly, subjects homozygous for the *FTO rs9939609* AA allele have dysregulated orexigenic hormone acyl-ghrelin within brain regions that regulate appetite; thus, modulating the neural responses to food images in homeostatic and brain reward regions as evidenced by functional MRI. Furthermore, overexpression of *FTO* in cell models reduces methylation of ghrelin mRNA N^6^-methyladenosine, leading to increased ghrelin mRNA and peptide levels. The effect was also shown in the blood of AA subjects [45].

In addition to the central effect, *FTO* variants may exert an effect on cellular metabolism. The *rs9939609* is in linkage disequilibrium with *rs1421085* (T>C), which may lead to obesity through the disruption of AR1D5B- mediated repression of Irx3 and Irx5. This leads to a shift from browning to whitening programs in the mitochondria with reduced mitochondrial thermogenesis[46]. A direct interaction exists between the promoters of Iroquois homeobox gene 3 (*Irx3*) and the *FTO* in humans (and other species). Up to 30% weight loss may be due to genetic deficiency in *Irx3* Thus, *Irx3* is a key determinant of body mass and composition, probably by its interaction with *FTO* [47]. Interestingly, the partial deletion of *Irx3* in the hypothalamus may lead to an opposite effect [48]. The interaction between *Irx3* and *FTO* may vary according to the genotype and explain the effect on appetite [49].

*The FTO* interacts with several other proteins. To achieve full validity of the enrichment test, we added an entire set of proteins to the STRING interactive database, with 'first shell' and 'second shell' are both set to 'none' in the Data Setting box (protein-protein interaction ‘PPI’ enrichment, p-value = 0.0111). This lowered down the PPI enrichment p-value:< 1.0e^-16^. The *FTO* and Melanocortin receptor 4 (*MCR4*) are co-expressed in other species, but not in humans. The *MCR4* plays a central role in energy homeostasis and somatic growth. The *FTO* is also co-expressed with *ALKBH2*, another DNA oxidative demethylase [50].

The UAE is located at a geographic hub between Africa, Europe and Asia and was thus exposed to human dispersal waves (e.g. the Paleolithic "Out of Africa" migrations and the exodus of Neolithic pastoral agriculturalists from the Fertile Crescent and Northern Africa around 11,000 years ago [51]. UAE population is genetically highly heterogeneous [52]. Genetic characteristics of Emiratis are in common with the rest of Arabian Peninsula populations [53]. However, the Emirati population has a relatively high Asian component due to admixture with immigrants from geographically close countries [54]. Following an initial pilot study, it was feasible to recruit subjects from the University of Sharjah and primary health care centers to include variable age groups. University students and visitors attending the primary health care centers come from all over the country, although mainly from the city of Sharjah. This may be a limitation to our study, as it may not equally represent Emirati population from various backgrounds, nevertheless, the study includes a good representation of indigenous Emirati population.

This study is first of its kind to explore the effect of *FTO* SNPs on food predilection in the Emirati population. It showed interesting interactions among the two SNPs notorious for their link to obesity. In the future, we would like to replicate our results on an independent large cohort of subjects.

## Conclusion

The *FTO* genotype plays a significant role in determining the predilection and preference of macro- and micronutrients. The results of the current study highlight the effect of the *FTO* risk alleles interaction on Emiratis’ food intake. In contrast to previous studies in other ethnicities, we showed that the *FTO rs9939609* AA subjects have higher carbohydrate and a trend of lower fat intake. The latter is accentuated in presence of *rs9930506* GG genotype. Further investigations are required to elucidate potential interactions of SNPs and food preference, and to unleash the mechanistic link.

## Supporting information

S1 File Genotyping of study population. The file includes genotyping of two SNPs saved as *.sav file.
(SAV)

S2 File Food item intake of study population. The file includes daily intake of different food items saved as *.xl file.
(XLS)

## References

[1] Low S, Chin MC, Deurenberg-Yap M. Review on epidemic of obesity. Ann Acad Med Singapore. 2009;38: 57–9. Available: http://www.ncbi.nlm.nih.gov/pubmed/19221672 19221672

[2] Castillo JJ, Orlando RA, Garver WS. Gene-nutrient interactions and susceptibility to human obesity. Genes Nutr. 2017;12: 29 10.1186/s12263-017-0581-3 29093760 PMC5663124

[3] Bouchard C. Childhood obesity: are genetic differences involved? Am J Clin Nutr. 2009;89: 1494S–1501S. 10.3945/ajcn.2009.27113C 19261728 PMC2677002

[4] Elks CE, den Hoed M, Zhao JH, Sharp SJ, Wareham NJ, Loos RJF, et al Variability in the Heritability of Body Mass Index: A Systematic Review and Meta-Regression. Front Endocrinol (Lausanne). 2012;3 10.3389/fendo.2012.00029 22645519 PMC3355836

[5] Christiansen E, Swann A, Sørensen TIA. Feedback models allowing estimation of thresholds for self-promoting body weight gain. J Theor Biol. 2008;254: 731–736. 10.1016/j.jtbi.2008.07.004 18671981

[6] Robinson MR, Hemani G, Medina-Gomez C, Mezzavilla M, Esko T, Shakhbazov K, et al Population genetic differentiation of height and body mass index across Europe. Nat Genet. 2015;47: 1357–1362. 10.1038/ng.3401 26366552 PMC4984852

[7] Loos RJF, Yeo GSH. The bigger picture of FTO—the first GWAS-identified obesity gene. Nat Rev Endocrinol. 2014;10: 51–61. 10.1038/nrendo.2013.227 24247219 PMC4188449

[8] Hunt SC, Stone S, Xin Y, Scherer CA, Magness CL, Iadonato SP, et al Association of the FTO Gene With BMI. Obesity. 2008;16: 902–904. 10.1038/oby.2007.126 18239580 PMC4476623

[9] Frayling TM, Timpson NJ, Weedon MN, Zeggini E, Freathy RM, Lindgren CM, et al A Common Variant in the FTO Gene Is Associated with Body Mass Index and Predisposes to Childhood and Adult Obesity. Science (80-). 2007;316: 889–894. 10.1126/science.1141634 17434869 PMC2646098

[10] Cyrus C, Ismail MH, Chathoth S, Vatte C, Hasen M, Al Ali A. Analysis of the Impact of Common Polymorphisms of the FTO and MC4R Genes with the Risk of Severe Obesity in Saudi Arabian Population. Genet Test Mol Biomarkers. 2018;22: 170–177. 10.1089/gtmb.2017.0218 29466028

[11] A. A-S, S.A. A-B, M. K, D. T, O. A, R. A-T, et al Association of FTO rs9939609 with Obesity in the Kuwaiti Population: A Public Health Concern? Med Princ Pract. 2018; 10.1159/000486767 29402776 PMC5968256

[12] Khan SM, El HajjChehadeh S, Abdulrahman M, Osman W, Al Safar H. Establishing a genetic link between FTO and VDR gene polymorphisms and obesity in the Emirati population. BMC Med Genet. 2018;19 10.1186/s12881-018-0522-z 29343214 PMC5773046

[13] Sabarneh A, Ereqat S, Cauchi S, AbuShamma O, Abdelhafez M, Ibrahim M, et al Common FTO rs9939609 variant and risk of type 2 diabetes in Palestine. BMC Med Genet. 2018;19: 156 10.1186/s12881-018-0668-8 30170548 PMC6119238

[14] Khella MS, Hamdy NM, Amin AI, El-Mesallamy HO. The (FTO) gene polymorphism is associated with metabolic syndrome risk in Egyptian females: a case- control study. BMC Med Genet. 2017;18: 101 10.1186/s12881-017-0461-0 28915859 PMC5603034

[15] Scuteri A, Sanna S, Chen WM, Uda M, Albai G, Strait J, et al Genome-wide association scan shows genetic variants in the FTO gene are associated with obesity-related traits. PLoS Genet. 2007;3: 1200–1210. 10.1371/journal.pgen.0030115 17658951 PMC1934391

[16] Radwan Hadia, Ballout Rami A., Hasan Hayder, Lessan Nader, Karavetian Mirey and RR. The Epidemiology and Economic Burden of Obesity and Related Cardiometabolic Disorders in the United Arab Emirates: A Systematic Review and Qualitative Synthesis. J Obes. 2018; 10.1155/2018/2185942 30652030 PMC6311818

[17] Ng SW, Zaghloul S, Ali HI, Harrison G, Popkin BM. The prevalence and trends of overweight, obesity and nutrition-related non-communicable diseases in the Arabian Gulf States. Obes Rev. 2011;12: 1–13. 10.1111/j.1467-789X.2010.00750.x 20546144

[18] World Health Organization (WHO). Obesity: Preventing and Managing the Global Epidemic. WHO Tech Rep Ser. 2000; doi:ISBN 92 4 120894 5 11234459

[19] Naja F, Nasreddine L, Itani L, Chamieh MC, Adra N, Sibai AM, et al Dietary patterns and their association with obesity and sociodemographic factors in a national sample of Lebanese adults. Public Health Nutr. 2011;14: 1570–1578. 10.1017/S136898001100070X 21557871

[20] Saber-Ayad M, Manzoor S, El Serafi A, Mahmoud I, Hammoudeh S, Rani A, et al The FTO rs9939609 “A” allele is associated with impaired fasting glucose and insulin resistance in Emirati population. Gene. 2019;681: 93–98. 10.1016/j.gene.2018.09.053 30273662

[21] Rodriguez S, Gaunt TR, Day INM. Hardy-Weinberg Equilibrium Testing of Biological Ascertainment for Mendelian Randomization Studies. Am J Epidemiol. 2009;169: 505–514. 10.1093/aje/kwn359 19126586 PMC2640163

[22] Locke A, Kahali B, Berndt S, Justice A, Pers T. Genetic studies of body mass index yield new insights for obesity biology. Nature. 2015;518: 197–206. 10.1038/nature14177 25673413 PMC4382211

[23] Shabana, Hasnain S. Effect of the Common Fat Mass and Obesity Associated Gene Variants on Obesity in Pakistani Population: A Case-Control Study. Biomed Res Int. 2015;2015: 1–8. 10.1155/2015/852920 26357660 PMC4555445

[24] Baturin AK, Sorokina EIu, Pogozheva AV, Anokhina OV TV. The study of FTO rs9939609-gene polymorphism in the Sverdlovsk Region. Vopr Pitan. 2012;81: 28–32. 23461169

[25] Li H, Wu Y, Loos RJ, Hu FB, Liu Y, Wang J, et al Variants in the fat mass- and obesity-associated (FTO) gene are not associated with obesity in a Chinese Han population. Diabetes. 2008;57: 264–268. 10.2337/db07-1130 17959933

[26] Jacobsson JA, Danielsson P, Svensson V, Klovins J, Gyllensten U, Marcus C, et al Major gender difference in association of FTO gene variant among severely obese children with obesity and obesity related phenotypes. Biochem Biophys Res Commun. 2008;368: 476–482. 10.1016/j.bbrc.2008.01.087 18249188

[27] Zhang M, Zhao X, Cheng H, Wang L, Xi B, Shen Y, et al Age- and Sex-Dependent Association between FTO rs9939609 and Obesity-Related Traits in Chinese Children and Adolescents. Li S, editor. PLoS One. 2014;9: e97545 10.1371/journal.pone.0097545 24827155 PMC4020831

[28] Hallman DM, Friedel VC, Eissa MAH, Boerwinkle E, Huber JC, Harrist RB, et al The association of variants in the FTO gene with longitudinal body mass index profiles in non-Hispanic white children and adolescents. Int J Obes. 2012; 10.1038/ijo.2011.190 21986706 PMC3495000

[29] Young AI, Wauthier F, Donnelly P. Multiple novel gene-by-environment interactions modify the effect of FTO variants on body mass index. Nat Commun. 2016;7: 12724 10.1038/ncomms12724 27596730 PMC5025863

[30] Liu J, Tuvblad C, Raine A, Baker L. Genetic and environmental influences on nutrient intake. Genes Nutr. 2013;8: 241–252. 10.1007/s12263-012-0320-8 23055091 PMC3575882

[31] Malik VS, Popkin BM, Bray GA, Després JP, Hu FB. Sugar-sweetened beverages, obesity, type 2 diabetes mellitus, and cardiovascular disease risk. Circulation. 2010 10.1161/CIRCULATIONAHA.109.876185 20308626 PMC2862465

[32] Cecil JE, Tavendale R, Watt P, Hetherington MM, Palmer CNA, Ph D, et al An obesity-associated FTO gene variant and increased energy intake in children. N Engl J Med. 2008;359: 2558–2566. 10.1056/NEJMoa0803839 19073975

[33] Wardle J, Carnell S, Haworth CMA, Farooqi IS, O’Rahilly S, Plomin R. Obesity associated genetic variation in FTO is associated with diminished satiety. J Clin Endocrinol Metab. 2008;93: 3640–3643. 10.1210/jc.2008-0472 18583465

[34] Hebert JR, Ma Y, Clemow L, Ockene IS, Saperia G, Stanek EJ, et al Gender Differences in Social Desirability and Social Approval Bias in Dietary Self-report. Am J Epidemiol. 1997;146: 1046–1055. 10.1093/oxfordjournals.aje.a009233 9420529

[35] Sonestedt E, Roos C, Gullberg B, Ericson U, Wirfält E, Orho-Melander M. Fat and carbohydrate intake modify the association between genetic variation in the FTO genotype and obesity. Am J Clin Nutr. 2009;90: 1418–1425. 10.3945/ajcn.2009.27958 19726594

[36] Rosenquist JN, Lehrer SF, O’Malley AJ, Zaslavsky AM, Smoller JW, Christakis NA. Cohort of birth modifies the association between FTO genotype and BMI. Proc Natl Acad Sci. 2015; 10.1073/pnas.1411893111 25548176 PMC4299180

[37] Lourenço BH, Qi L, Willett WC, Cardoso MA. FTO genotype, vitamin D status, and weight gain during childhood. Diabetes. 2014;63: 808–814. 10.2337/db13-1290 24130335 PMC3900536

[38] Melhorn SJ, Askren MK, Chung WK, Kratz M, Bosch TA, Tyagi V, et al FTO genotype impacts food intake and corticolimbic activation. Am J Clin Nutr. 2018;107: 145–154. 10.1093/ajcn/nqx029 29529147 PMC6454473

[39] Fawcett KA, Barroso I. The genetics of obesity: FTO leads the way. Trends Genet. 2010;26: 266–274. 10.1016/j.tig.2010.02.006 20381893 PMC2906751

[40] Doo M, Kim Y. Obesity: Interactions of Genome and Nutrients Intake. Prev Nutr Food Sci. 2015;20: 1–7. 10.3746/pnf.2015.20.1.1 25866743 PMC4391534

[41] Akram DS, Astrup A V, Atinmo T, Boissin JL, Bray GA, Carroll KK, et al Obesity: Preventing and managing the global epidemic. World Health Organization—Technical Report Series 2000. 11234459

[42] Giskes K, van Lenthe F, Avendano-Pabon M, Brug J. A systematic review of environmental factors and obesogenic dietary intakes among adults: are we getting closer to understanding obesogenic environments? Obes Rev. 2011;12: e95—e106. 10.1111/j.1467-789X.2010.00769.x 20604870

[43] Zhou J, Wan J, Gao X, Zhang X, Jaffrey SR, Qian S-B. Dynamic m6A mRNA methylation directs translational control of heat shock response. Nature. 2015;526: 591–594. 10.1038/nature15377 26458103 PMC4851248

[44] Berulava T, Horsthemke B. The obesity-associated SNPs in intron 1 of the FTO gene affect primary transcript levels. Eur J Hum Genet. 2010;18: 1054–1056. 10.1038/ejhg.2010.71 20512162 PMC2987405

[45] Karra E, O’Daly OG, Choudhury AI, Yousseif A, Millership S, Neary MT, et al A link between FTO, ghrelin, and impaired brain food-cue responsivity. J Clin Invest. 2013;123: 3539–3551. 10.1172/JCI44403 23867619 PMC3726147

[46] Claussnitzer M, Dankel SN, Kim K-H, Quon G, Meuleman W, Haugen C, et al FTO Obesity Variant Circuitry and Adipocyte Browning in Humans. N Engl J Med. 2015;373: 895–907. 10.1056/NEJMoa1502214 26287746 PMC4959911

[47] Smemo S, Tena JJ, Kim KH, Gamazon ER, Sakabe NJ, Gómez-Marín C, et al Obesity-associated variants within FTO form long-range functional connections with IRX3. Nature. 2014;507: 371–375. 10.1038/nature13138 24646999 PMC4113484

[48] TM de Araujo DS Razolli FC-S. The partial inhibition of hypothalamic IRX3 exacerbates obesity. EBioMed. 2018;in print. 10.1016/j.ebiom.2018.11.048 PMC6354701 30522931

[49] Schneeberger M. Irx3, a new leader on obesity genetics. EBioMed. 2018;in print. 10.1016/j.ebiom.2018.12.005 PMC6354703 30541683

[50] Szklarczyk D, Morris JH, Cook H, Kuhn M, Wyder S, Simonovic M, et al The STRING database in 2017: quality-controlled protein–protein association networks, made broadly accessible. Nucleic Acids Res. 2017;45: D362–D368. 10.1093/nar/gkw937 27924014 PMC5210637

[51] Nielsen R, Akey JM, Jakobsson M, Pritchard JK, Tishkoff S, Willerslev E. Tracing the peopling of the world through genomics. Nature. 2017;541: 302–310. 10.1038/nature21347 28102248 PMC5772775

[52] Al-Ali M, Osman W, Tay GK, AlSafar HS. A 1000 Arab genome project to study the Emirati population. J Hum Genet. 2018;63: 533–536. 10.1038/s10038-017-0402-y 29410509 PMC5867278

[53] Garcia-Bertrand R, Simms TM, Cadenas AM, Herrera RJ. United Arab Emirates: Phylogenetic relationships and ancestral populations. Gene. 2014;533: 411–419. 10.1016/j.gene.2013.09.092 24120897

[54] Al-Gazali L, Ali BR. Mutations of a country: a mutation review of single gene disorders in the United Arab Emirates (UAE). Hum Mutat. 2010;31: 505–520. 10.1002/humu.21232 20437613

